# ‘Humour-ability’ of mental health professionals

**DOI:** 10.4103/0019-5545.31567

**Published:** 2006

**Authors:** Nimesh G. Desai, S.N. Sengupta, Deepak Kumar, Rupali Shivalkar, Saurabh Mehrotra

**Affiliations:** * Professor, Institute of Human Behaviour and Allied Sciences (IHBAS), Dilshad Garden, Delhi 110095; ** Professor, Institute of Human Behaviour and Allied Sciences (IHBAS), Dilshad Garden, Delhi 110095; *** Assistant Professor, Institute of Human Behaviour and Allied Sciences (IHBAS), Dilshad Garden, Delhi 110095; **** Assistant Professor, Institute of Human Behaviour and Allied Sciences (IHBAS), Dilshad Garden, Delhi 110095; ***** Senior Resident, Institute of Human Behaviour and Allied Sciences (IHBAS), Dilshad Garden, Delhi 110095

Compliments to Dr Swaminath^1^ for having thought of and highlighting such a novel theme for mental health professionals. Humour is humane, humour brings two minds closer, humour dissociates one from the prangs of immediate experience. Naturally, the relevance of such a high order human faculty or the ‘invisible weapon’ ordained by the Lord for the mankind, especially in mental health, cannot be questioned. No wonder, only a person like Dr Swaminath could write so glibly on this topic. Whoever has worked or come in contact with him can never miss his ‘free’ humourous gift of the gab and an infectious spirit of jocularity. While the article makes a wonderful reading, one cannot miss the implicit finger pointing to those working in mental health as to remind us how much sensitized are we to this issue and translate that in in our day-to-day clinical work.

The question is not easy to answer, although we may agree with Dr Swaminath that humour is therapeutic and serves a number of salutary functions for the individual and groups. Several factors influence the ability to use humour by an individual as a member of society or as a mental health professional. ‘Humour-ability’ is, by and large, part of one's personality. People with different personal attributes perceive humour differently. One is bound to a great extent by the way one thinks or feels for others, one's own psychological makeup and that being, in turn, dependent on the inherited neuronal circuits and early experiences. The sense of humour is a special ability endowed to the human beings and cultivation of the same is no laughing matter. Yet, one can try to learn and adapt if one so desires.

Some may have the natural gift of making an apt use of humour in all kinds of interactions. Others may only be able to appreciate the use of it but may fall short of using it on a right niche of conversation; still some may well laugh with a joke on others but may feel awkward to have it on self. In fact sense of humour is related to the facet of fantasy on the dimension of ‘openness to experience’ in the popular Five Factor Model of Personality^2^ and probably goes with the component of self transcendence in the character dimensions of Temperament Character Inventory^3^. As a leading psychiatrist Warren Poland writes, ‘Humour… reflects a regard for oneself and one's limits, despite pain. With such humour there is an acceptance for what one is…’^4^ The ability to laugh at oneself is not an easy task though not impossible either. There is a need to learn to laugh at oneself as an individual and also as a professional, while not being burdened by stigma.

The general notion is that sense of humour is to be better preserved for informal social interactions and it is often considered a taboo to crack even half a smile before a patient. Therapist–patient relationship is viewed as a professional one and so the therapist is bound to be wary of any inept behaviour during such interactions. The medicos trained in western model of medicine tend to uphold a subculture of putting on a serious and somber attitude towards the clients. Doctors naturally strive to wear a look of seriousness and suppress cheerfulness and jocularity in their professional demeanour. The work and the movie on *Patch Adams* and its Indian adaptation ‘Munnabhai MBBS’ have questioned the physician's impersonal approach to their patients. The films are relevant to both the practice of medicine as well as psychiatry. Though the burden of responsibility of saving a patient rests on the physician's shoulders, humour might help to lighten it.

Humour is of several types: healthy, comic, cynical/satire, witty, slapstick/silly or even sexual. The ability to distinguish healthy humour from cynical which is at the expense of others' shortcomings is important. The *shastra* (tool) of humour needs to be used discreetly so as not to hurt the other person. Notwithstanding all the complex issues and skepticism, clinicians would do well to use humour as a visiting card as suggested by Dr Swaminath.
